# The Maternal and Fetal Consequences of Metabolic Dysfunction-Associated Fatty Liver Disease and Gestational Diabetes Mellitus

**DOI:** 10.3390/nu17101730

**Published:** 2025-05-20

**Authors:** Thora Y. Chai, Jacob George, Dharmintra Pasupathy, Ngai Wah Cheung, Victoria L. Rudland

**Affiliations:** 1 Macarthur Diabetes Service, Campbelltown Hospital, Campbelltown, NSW 2560, Australia; 2 Faculty of Medicine and Health, The University of Sydney, Sydney, NSW 2006, Australia; jacob.george@sydney.edu.au (J.G.); dharmintra.pasupathy@sydney.edu.au (D.P.); wah.cheung@sydney.edu.au (N.W.C.); victoria.rudland@sydney.edu.au (V.L.R.); 3 Reproduction and Perinatal Centre, Faculty of Medicine and Health, Westmead Hospital, The University of Sydney, Westmead, NSW 2145, Australia; 4 Storr Liver Centre, Westmead Millennium Institute for Medical Research, Westmead Hospital and The University of Sydney, Westmead, NSW 2145, Australia; 5 Department of Gastroenterology and Hepatology, Westmead Hospital, Westmead, NSW 2145, Australia; 6 Department of Diabetes and Endocrinology, Westmead Hospital, Westmead, NSW 2145, Australia

**Keywords:** metabolic dysfunction-associated fatty liver disease, MAFLD, gestational diabetes mellitus, GDM, pregnancy, maternal outcome, neonatal outcomes

## Abstract

Both metabolic dysfunction-associated fatty liver disease (MAFLD) and gestational diabetes mellitus (GDM) during pregnancy are emerging as an adverse synergistic relationship of growing concern. This narrative review focuses on the maternal and fetal consequences associated with women who have MAFLD and/or GDM during pregnancy, including an exploration of long-term cardiometabolic risks for postpartum maternal and childhood health. We conclude that implementation of a life course approach to management of these high-risk women remains paramount.

## 1. Introduction

Metabolic dysfunction-associated fatty liver disease (MAFLD), formerly known as non-alcoholic fatty liver disease (NAFLD), is one of the most prevalent chronic liver diseases worldwide [1,2,3,4,5]. As a hepatic manifestation of the metabolic syndrome, MAFLD rates have increased in concert with those of other associated metabolic disorders, including obesity, hypertension, dyslipidaemia and type 2 diabetes (T2D) [1,2,3,4,5]. While the NAFLD and MAFLD terms refer to slightly different entities, with a Cohen’s kappa of up to 0.92 [6], the overall concordance of the two definitions is high, up to 100% [6,7]. In this review, the term MAFLD will be used when referring to this entity, unless otherwise stated.

Alarmingly, a recent study indicated that the prevalence of MAFLD has increased in the young adult population, which has implications for pregnancy [8]. Pregnancy data confirm a tripling of MAFLD prevalence during pregnancy over the last 10 years [9]. The presence of MAFLD has been associated with increased adverse maternal and neonatal outcomes [9,10,11,12,13,14]. In particular, an adverse bidirectional relationship appears to exist between MAFLD and gestational diabetes mellitus (GDM), defined as glucose intolerance first detected during pregnancy [15]. GDM is also escalating globally in the context of increasing rates of T2D and obesity [15,16]. Both MAFLD and GDM during pregnancy appear to not only lead to shorter-term pregnancy complications [9,17,18,19,20], but also longer-term cardiometabolic complications for both the mother and their offspring, given that both MAFLD and GDM are metabolic conditions that individually predispose women to a higher risk of cardiovascular events [21,22,23,24,25].

This narrative review will explore and synthesise the evidence behind MAFLD and GDM, and provide an overview of both their individual and synergistic consequences on maternal and fetal health during and after pregnancy. Possible management strategies in this growing, yet under-recognised, clinical phenomena in at-risk reproductive aged women will also be discussed.

## 2. Methodology

The electronic databases of MEDLINE (Ovid) and PubMed were searched to identify relevant studies written in English and involving human studies only, published up to 28 February 2025. The databases were queried using the search terms “metabolic dysfunction-associated fatty liver disease”, “metabolic associated fatty liver disease”, “MAFLD”, “metabolic dysfunction-associated steatotic liver disease”, “metabolic associated steatotic liver disease”, “MASLD”, “non-alcoholic fatty liver disease”, “NAFLD”, “gestational diabetes mellitus”, “GDM”, “pregnancy”, “adverse maternal outcomes”, “adverse neonatal outcomes” and “adverse pregnancy outcomes”. The Boolean operator of and/or was used to combine the terms. A manual search was performed on the selected articles to identify other relevant studies from the reference lists of relevant articles and any articles known to the authors relevant to the topic and not identified through the search of the electronic databases.

## 3. Metabolic Dysfunction-Associated Fatty Liver Disease (MAFLD)

### 3.1. Prevalence of MAFLD

Currently, MAFLD is the most common liver disease worldwide [2,3,4,5,26,27,28]. It affects a third of the global adult population, with the highest prevalence rates occurring in the South Americas and the Middle East (around 30%) [2,3,4,5,28]. Markov modelling studies based on the liver ultrasound results collected from the Third National Health and Nutrition Examination Survey (NHANES III) data performed in the United States of America (USA) projected that MAFLD cases would increase by up to 11%, from 66.6 million in 2015 to 73.9 million in 2030 [29].

### 3.2. Risk Factors for MAFLD

A large driver in the rapid rise of MAFLD is its frequent coexistence with other metabolic conditions, particularly overweight and obesity [2,3,4,5]. The proportional increase in MAFLD prevalence rates with obesity is pertinent, given that the prevalence of obesity has risen 6-fold over the last four decades, particularly in adult women [3,4,5,30]. From 1975 to 2016, the number of adult women with obesity globally increased from 69 (57–83) million to 390 (363–418) million [30].

T2D is another important risk factor for the development of MAFLD [3,4,5], with a 2019 systematic review of 49,419 individuals with T2D from 20 countries determining that the overall global prevalence of MAFLD amongst those diagnosed with T2D was 55.5% (95% confidence interval [31] 47.3–63.7%) [32]. Concerningly, the presence of T2D can accelerate the course of MAFLD and is an independent predictor of advanced hepatic fibrosis and higher overall mortality [3,4,5,32].

### 3.3. Diagnosis of MAFLD

**Figure 1:**
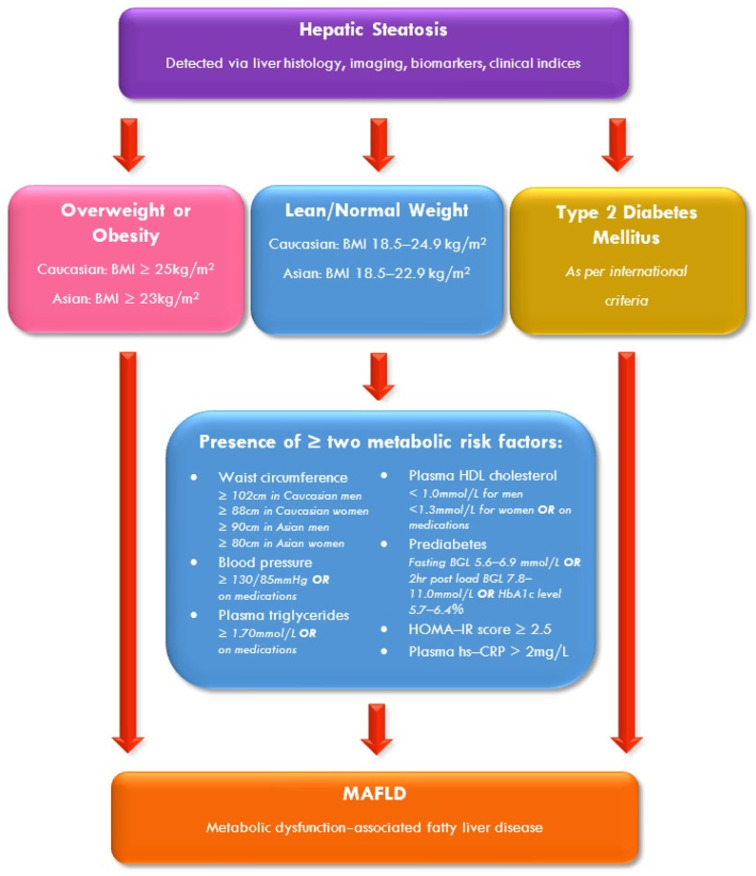
Diagnostic criteria for MAFLD. BGL: blood glucose level; BMI: body mass index; HDL: high-density lipoprotein; HOMA–IR: homeostatic model assessment for insulin resistance; hr: hour; hs–CRP: high-sensitivity C-reactive protein; MAFLD: metabolic dysfunction-associated fatty liver disease. Modified from Eslam et al. with permission [1].

MAFLD, unlike NAFLD, comes with positive diagnostic criteria, as illustrated in Figure 1. The diagnosis is based on the presence of hepatic steatosis (detected via non-invasive methods of imaging or clinical biomarkers, or invasive liver histology) in the context of either obesity, T2D or in persons of healthy weight, the presence of at least two risk factors that suggest metabolic dysregulation [1].

### 3.4. Management of MAFLD

The cornerstone of management of MAFLD centres on lifestyle interventions [5,33,34]. Improved nutrition plays a major role in the management of MAFLD, where adherence to a Mediterranean, low-carbohydrate (<60 g of carbohydrate per day) or low-calorie (>500 calorie deficit per day) diet helped to reduce the severity of MAFLD, or even led to its remission, mostly by reducing body weight and thereby hepatic steatosis [5,33,34,35,36]. However, the Mediterranean diet, characterised by reduced intake of refined carbohydrates, saturated fats and red, processed meats and increased intake of fruits, vegetables, wholegrains, legumes, nuts/seeds and olive oil, is currently recommended by the European Association for the Study of Liver (EASL), European Association for the Study of Diabetes (EASD) and European Association for the Study of Obesity (EASO) 2024 Guidelines, as it was considered a diet easier to sustain and had significant advantages of reducing hepatic lipid levels as well as improving long-term cardiometabolic health [5]. The use of specific nutrient supplements, or nutraceuticals, in the management of MAFLD remains controversial. However, vitamin E supplementation of 800IU per day has been recommended by the EASL-EASD-EASO 2024 Guidelines [5]. Previously, a large, randomised controlled trial (RCT) identified that vitamin E at 800IU daily taken over 2 years’ duration helped to significantly reduce hepatic steatosis [37].

In addition to dietary modifications, moderate-intensity aerobic exercise for 150 min per week, or high-intensity exercise for 100 min per week, significantly helped to reduce hepatic steatosis. This finding was independent of associated weight loss. As such, regular exercise is recommended for all people with MAFLD, unless contraindications to intense exercise exist [5,33,34]. In those with MAFLD and obesity, gradual body weight loss of even 3–5% is beneficial in reversing hepatic steatosis, so is highly recommended [5,33,34]. Even more encouraging, greater body weight loss of 7–10% can reduce hepatic steatosis further by at least 45–50% and also helps to improve hepatic inflammation, but not hepatic fibrosis, until weight loss of ≥10% is achieved [5,34].

## 4. Gestational Diabetes Mellitus (GDM)

### 4.1. Prevalence of GDM

With the global prevalence of GDM exponentially increasing, it is fast becoming one of the most common medical complications experienced by women in pregnancy [15,38]. The International Diabetes Federation (IDF) estimated in 2025 that in women aged 20–49 years, 23 million (19.7%) live births were affected by GDM [15]. Based on the International Association of Diabetes and Pregnancy Study Groups (IADPSG) criteria, the global prevalence of GDM is estimated to be 14.0% [39]. If GDM is diagnosed earlier, i.e., prior to 20 weeks’ gestation, the global prevalence ranged between 0.7 and 36.8%, irrespective of the diagnostic criteria used [39,40].

### 4.2. Risk Factors for GDM

Advanced maternal age, defined as pregnancy in women over 35 years [41,42], is associated with a higher risk of GDM, with women over 40 years experiencing a 2-fold increased risk compared with women aged between 25 and 30 years [43]. Certain ethnicities are more susceptible to developing GDM, with extremely high-risk ethnicities including Pacific Islanders, Hispanic, Australian Indigenous, Southeast Asian and South Asian, whilst ethnicities considered at moderate risk include Middle Eastern, Northern African, African American and East Asian [44,45,46]. Other significant risk factors for GDM and its recurrence include a familial predisposition towards T2D, particularly having a first-degree relative affected, a diagnosis of polycystic ovarian syndrome (PCOS), a prior history of GDM and the presence of macrosomia (defined as neonatal birth weight over 4000 g, or neonatal birthweight > 90th percentile) [47,48,49,50,51].

Finally, maternal weight prior to pregnancy is another important independent predictor for the development of GDM, with the risk of GDM directly related to maternal body mass index (BMI) [43,51,52]. It has been estimated that even a modest rise in weight of 5.0–9.9 kg from 18 years of age until onset of index pregnancy can result in a 2-fold increased risk of GDM [43]. This risk is exponentially amplified in women who are overweight or obese prior to pregnancy [51,53].

### 4.3. Diagnosis of GDM

Currently, the diagnosis of GDM is determined through universal screening of all pregnant woman with the oral glucose tolerance test (OGTT) [38]. The IADPSG recommends screening for GDM at 24–28 weeks’ gestation with a one-step, 2 h 75 g OGTT [54]. The diagnosis of GDM is made if any one of the three OGTT blood glucose levels (BGL) equals or exceeds the diagnostic thresholds of fasting BGL of 5.1 mmol/L, 1 h BGL of 10.0 mmol/L and/or 2 h BGL of 8.5 mmol/L [54]. These diagnostic thresholds were chosen based on the IADPSG panel consensus, as they corresponded with a 1.75-fold increased risk of adverse pregnancy outcomes in the Hyperglycaemia and Adverse Pregnancy Outcome (HAPO) study [54,55,56]. Although the IADPSG diagnostic criteria were established in 2010, global uptake of these criteria remains mixed [55].

### 4.4. Management of GDM

The essential components in the management of GDM include medical nutrition therapy, physical activity and self-monitoring of blood glucose (SMBG) [39,57]. The recommended minimum nutritional intake for women during pregnancy includes 175 g of carbohydrate, 71 g of protein and 28 g of fibre [57]. Carbohydrate restriction is not recommended in the management of GDM, because adequate caloric intake is required for fetal health and development [57]. Thus, promoting the intake of higher-quality, low-glycaemic-index carbohydrates is preferred for women with GDM in order to ensure they meet glycaemic targets, reduce insulin resistance and excess neonatal/infant adiposity [57].

In women with uncomplicated pregnancies, 150 min per week of moderate-intensity aerobic and strength-conditioning exercise is recommended to reduce the risk of excessive gestational weight gain [57]. Physical activity recommendations for women with GDM include 20–50 min per day of moderate-intensity aerobic, resistance or both types of exercises for at least 5–7 days a week [57]. Most importantly, women with GDM are encouraged not to remain sedentary during the duration of their pregnancy, as most studies positively indicate that over 70% of women with GDM are able to manage with lifestyle modification alone [39,57].

Regular SMBG is essential in the management of GDM, and involves women checking their fingerprick glucose four times a day at predetermined intervals, which are commonly fasting and 2 h after the start of each of the three main meals [38,39,57]. Guideline target glucose levels remain variable in different countries [38,39,57]. However, two large RCTs identified the benefits of treating GDM to strict glycaemic targets, specifically fasting glucose levels of <5.3 or <5.5 mmol/L, and 2 h postprandial glucose levels of <6.7 or <7.0 mmol/L [58,59]. Ideally, SMBG is reviewed by the healthcare practitioner at each clinic visit and helps to determine whether pharmacotherapy is necessary [39,57]. If glucose levels are above the glycaemic targets for GDM without adequate dietary or lifestyle explanation (i.e., not due to excess carbohydrate intake or lack of physical activity), the intensification of GDM treatment with medication is required [38,39,57]. Preferred medications to use in women with GDM would be either insulin therapy and/or metformin. However, metformin can be placentally transferred, with long-term fetal effects as yet unknown, and therefore its use during a GDM-complicated pregnancy is controversial. Specifically, the use of metformin in women with GDM and small-for-gestational age (SGA) neonates is not advised [38,39,57].

In terms of the obstetric management of GDM, close monitoring of fetal growth in women with GDM is essential, as it helps to determine subsequent delivery plans (i.e., induction of labour +/− caesarean delivery) [38,39]. Serial fetal growth ultrasounds performed between 28 and 36 weeks of gestation in women with GDM, which assess fetal abdominal circumference and estimated fetal weights, have been shown to be useful in determining delivery plans and further guiding the medical management of GDM [38,39]. However, the optimal timing and mode of delivery in women with GDM are complex and should remain individualised [38,39].

In terms of postpartum care, anti-hyperglycaemic agent therapy is usually discontinued after delivery [38,39,57]. Neonates of women with GDM are often monitored for neonatal hypoglycaemia with heel-prick glucose monitoring, and if present, require short-term management in high-dependency neonatal wards [38,39]. Women with GDM are advised to perform follow-up glucose testing with a 75 g OGTT using non-pregnancy diagnostic criteria about 4–12 weeks postpartum to screen for T2D [38,39,57].

## 5. The Relationship Between Metabolic Dysfunction-Associated Fatty Liver Disease and Gestational Diabetes Mellitus

To date, the relationship between MAFLD and GDM appears bidirectional, and although limited studies are available, the presence of both MAFLD and GDM likely leads to adverse synergistic consequences.

### 5.1. A Prior History of GDM Predicts the Postpartum Development of MAFLD

A summary of existing studies on postpartum MAFLD development in women with a prior history of GDM is outlined in Table 1.

Forbes et al. [60] studied the prevalence of, and risk factors for, MAFLD in a largely Caucasian cohort of women with a prior history of GDM [60]. They determined that ultrasound-detected MAFLD less than 10 years after the index pregnancy was significantly higher in women with a prior history of GDM compared to those with normoglycaemia (38% vs. 17%, *p* = 0.001). When adjusted for postpartum BMI, the odds ratio (OR) for the development of MAFLD remained doubled in women with a prior history of GDM (Table 1), suggesting an independent association [60].

To a lesser extent, Foghsgaard et al. [61] replicated this association between MAFLD development in women with a prior history of GDM [61]. In their cohort of 111 mostly Caucasian women, 24 (22%) developed MAFLD as detected on liver ultrasound [61]. The presence of MAFLD was independently associated with increasing insulin resistance, calculated according to the Matsuda Index (*p* = 0.006), and a larger waist circumference (*p* = 0.011), further reaffirming the known relationship between MAFLD, obesity and insulin resistance [61]. Given their smaller sample size, Foghsgaard et al. [61] were not able to determine any difference in the prevalence of prediabetes between women with and without MAFLD [61].

With a larger sample size of 257 women, Mehmood et al. [62] demonstrated that MAFLD was more prevalent in women with a prior history of GDM compared to those without (*p* = 0.009) [62]. MAFLD was also an independent predictor of prediabetes or T2D development at 5 years postpartum in women with a prior history of GDM (Table 1) [62].

Longer-term follow-up studies, such as the Coronary Artery Risk Development in Young Adults (CARDIA) cohort study in 2016 by Ajmera et al. [63], continued to demonstrate the strong association of postpartum MAFLD development in women with a prior history of GDM [63]. In a biracial cohort (57% African American and 43% Caucasian), computed tomography (CT)-detected MAFLD (using a cut-off liver attenuation value ≤ 40 Hounsfield unit [HU]) was significantly higher in women with a prior history of GDM compared to those without at 25 years follow-up (14% vs. 5.8%; unadjusted OR 2.56, 95% CI 1.44–4.55, *p* < 0.01) [63]. After adjustment for age, parity, BMI, waist circumference, homeostatic model assessment for insulin resistance (HOMA-IR), fasting triglyceride and high-density lipoprotein cholesterol (HDL-C) level, a prior history of GDM remained an independent predictor of postpartum MAFLD development, along with fasting triglyceride and HOMA-IR level (Table 1) [63]. However, a major limitation of this study is the absence of any baseline MAFLD screening, and thus the temporal relationship between MAFLD and a prior history of GDM may be attenuated by the possibility that MAFLD may have been present prior to, or at the time, of index pregnancy [63].

Finally, a large retrospective cohort study involving 64,397 predominantly Korean women (59,714 without a prior history of GDM and 4683 with a prior history of GDM, as determined through self-report) published by Cho et al. in 2023 [64] demonstrated that a prior history of GDM was an independent risk factor for overall MAFLD development, as determined by liver ultrasound (Table 1) [64]. Cho et al. [64] further investigated whether the effects of insulin resistance (as measured by HOMA-IR) or development of T2D were mediators of an association between a prior history of GDM and MAFLD, although this came at <10% association for both HOMA-IR and T2D [64], suggesting that the development of MAFLD in women with a history of GDM is complex and does not only involve insulin resistance.

### 5.2. The Association of MAFLD with the Development of GDM

Whether hepatic steatosis influences the subsequent development of GDM has not been extensively explored. Table 2 summarises the current studies examining the relationship between the antenatal detection of MAFLD and its association with the development of GDM.

There may be a significantly higher risk of GDM if MAFLD is detected during the first trimester of pregnancy. De Souza et al. [11] indicated that MAFLD identified during early pregnancy (11–14 weeks’ gestation) on liver ultrasound in a multiethnic cohort of 476 women with singleton pregnancies was associated with a higher occurrence of GDM in mid-pregnancy (24–28 weeks’ gestation) [11]. MAFLD remained an independent predictor of dysglycaemia (not only limited to GDM, but also impaired fasting glucose [IFG] and impaired glucose tolerance [IGT]) after adjustment for maternal age, ethnicity, first-degree relative with T2D, BMI (measured at 11–14 weeks’ gestation) and change in BMI readings between 11 and 14 weeks, and 24 and 28 weeks’ gestation (Table 2) [11].

In a largely Egyptian pregnancy cohort of 400 nulliparous women, Mousa et al. [65] again used liver ultrasound during the first trimester to demonstrate that a higher incidence of GDM occurred in women with MAFLD compared to those without (33% vs. 10%, *p* = 0.001) [65]. They also observed an increased occurrence of pre-eclampsia and gestational hypertension (blood pressure > 140/90 mmHg) manifesting in pregnant women with MAFLD over those without MAFLD (respectively, 25% vs. 14%, *p* = 0.001 and 7.5% vs. 2.5%, *p* = 0.013) [65]. A major limitation of this study was that only univariate analysis was performed, and no adjustment was made for possible confounders, including maternal age and other metabolic parameters.

Similar findings were also identified by Lee et al. [12] in their much larger longitudinal prospective study [12]. In their study population of 608 Korean women with singleton pregnancies, liver ultrasound was performed to detect MAFLD at 10–14 weeks’ gestation [12]. They identified 112 women (18.4%) with MAFLD, where 14.1% had mild/grade 1 hepatic steatosis and 4.3% had moderate/grade 2 or severe/grade 3 hepatic steatosis. The odds of developing GDM were 6.6 times higher (95% CI 3.36–13.4, *p* < 0.001) in women with MAFLD compared to those without (on age adjustment alone) [12]. When additionally adjusted for other metabolic risk factors, such as a prior history of GDM, larger waist circumference, HOMA-IR, higher systolic and diastolic blood pressure readings, plasma adiponectin and selenoprotein P levels, there remained a significant, although slightly attenuated, increased odds of GDM development (Table 2) [12]. This risk persisted in a stepwise fashion with increasing severity of MAFLD, as assessed by the Fatty Liver Index (FLI) and Hepatic Steatosis Index (HSI) [12]. Interestingly, regardless of MAFLD status, low maternal plasma adiponectin and high selenoprotein P levels were independent predictors of GDM development, suggesting that both biomarkers may be useful in predicting GDM and MAFLD [12].

Deng et al. [66] published a pilot study using FibroScan^®^ as the imaging modality to detect MAFLD in early-to-mid pregnancy (prior to 24 weeks’ gestation) [66]. In a multiethnic cohort, 6 (12%) of 50 pregnant women were diagnosed with MAFLD in early-to-mid pregnancy. Of these, only 3 (50%) women with FibroScan^®^-detected MAFLD in early pregnancy developed GDM compared to 13 (29.5%) women without MAFLD, which was not statistically significant (*p* = 0.37), likely due to the small sample size [66]. Another potential limitation of this study was the lack of validated FibroScan^®^ cut-off values for the controlled attenuation parameter (CAP) and liver stiffness measurement (LSM) specific to the pregnant population [66].

Two studies performed liver ultrasounds during the third trimester, after screening for, and diagnosis of, GDM was made at around 24–28 weeks’ gestation [67,68]. Herath et al. [67] determined in a largely South Asian (Sri Lankan) cohort of 573 pregnant women that MAFLD prevalence was 18.2%, and more women with MAFLD developed a composite of GDM and overt diabetes in pregnancy compared to those without (Table 2) [67]. The presence of MAFLD also led to a 2-fold increase in odds of developing maternal hypertensive complications, including gestational hypertension and pre-eclampsia, compared to those without MAFLD, even after adjustment for age, BMI and hyperglycaemia in pregnancy (aOR 2.09, 95% CI 1.07–4.10) [67].

In a smaller cohort of pregnant women of predominantly Caucasian ethnicity (*n* = 84), Sattari et al. [68] determined a similar MAFLD prevalence to Herath et al. [67] at 14.3% (*n* = 12) [68]. Given the small number of patients, they were unable to determine a statistically significant association between MAFLD and GDM (*p* = 0.21; Table 2), although MAFLD was only detected via liver ultrasound in the third trimester of pregnancy. It has been suggested that the detection of hepatic steatosis may be more sensitive during the third trimester, due to the increased insulin resistance occurring around this time [68,69]; however, the detection of MAFLD after screening for GDM makes it difficult to conclude whether MAFLD predisposes to GDM development.

Finally, our systematic review and meta-analysis of seven studies (total 2299 participants) demonstrated that women with MAFLD diagnosed via imaging studies conducted in pregnancy had a 3-fold increased risk of developing GDM compared with women without MAFLD (OR 2.9, 95% CI 1.0–8.4, *p* < 0.05) [70]. However, significant heterogeneity between the studies was present [70].

Indeed, most of the studies evaluating the relationship between a prior history of GDM and postpartum MAFLD development, and those evaluating the relationship between the detection of MAFLD in pregnancy and development of GDM, are observational in nature, performed in a single centre and typically with a limited sample size. Further research examining the relationship between MAFLD and GDM is required.

### 5.3. Short-Term Adverse Pregnancy Outcomes of MAFLD and GDM

The adverse maternofetal consequences of GDM are well documented. The HAPO study demonstrated an association between maternal hyperglycaemia in women with GDM and several neonatal complications, including macrosomia, shoulder dystocia, prematurity, fetal hyperinsulinaemia, neonatal hypoglycaemia, neonatal intensive care unit admissions and hyperbilirubinaemia [17]. Adverse maternal outcomes for women with GDM include increased rates of gestational hypertension, pre-eclampsia, primary caesarean delivery and perineal injuries requiring episiotomies, in comparison to women without GDM [17,18].

The exploration of the effects of MAFLD in pregnancy, including on maternal and fetal outcomes, has been limited until recent years. Two studies, one from Sweden [71] and the other from the USA [9], undertook retrospective analyses of their respective countries’ national patient databases to determine whether a diagnosis of MAFLD prior to pregnancy was associated with adverse maternal and/or neonatal outcomes. Hagstrom et al. [71] used the Swedish Medical Birth Register to identify women who had delivered over a 10-year period and linked their data with the Swedish National Patient Register to determine if a diagnosis of MAFLD (via the International Classification of Diseases [ICD] code for MAFLD) was present prior to delivery [71]. Out of a total of 1,960,416 singleton pregnancies, 110 women were diagnosed with MAFLD prior to delivery [71]. When women with MAFLD were compared to those without MAFLD or PCOS, they had a higher risk of GDM (adjusted risk ratio [aRR] 2.78, 95% CI 1.25–6.15), caesarean section (aRR 1.52, 95% CI 1.19–1.94), pre-eclampsia (aRR 1.95, 95% CI 1.03–3.70), prematurity (<37 weeks delivery; aRR 2.50, 95% CI 1.38–4.55), extreme prematurity (<32 weeks delivery; aRR 6.92, 95% CI 2.96–16.14) and low birth weight (aRR 2.40, 95% CI 1.21–4.78) [71].

Sarkar et al. [9] used discharge records from the USA 2007–2016 National Inpatient Sample (NIS) database [9]. Again, a diagnosis of MAFLD was identified prior to pregnancy using ICD codes, which were also used to diagnose adverse maternal (postpartum haemorrhage, maternal death during delivery, GDM and hypertensive pregnancy complications, including pre-eclampsia, eclampsia, haemolysis, elevated liver enzymes and low platelets [HELLP] syndrome) and neonatal (prematurity, intrauterine fetal growth restriction (IUGR), large-for-gestational age (LGA) neonate [72] and fetal death, including intrauterine or stillbirth) outcomes [9]. Alarmingly, a tripling in prevalence rates of MAFLD during pregnancy was identified across 2007–2016, increasing from 10.5/100,000 pregnancies to 28.9/100,000 pregnancies [9]. GDM, hypertensive pregnancy complications, postpartum haemorrhage and prematurity occurred more frequently in women with MAFLD diagnosed prior to pregnancy (*n* = 5640) compared to those with other chronic liver diseases (*n* = 115,210) or those with neither MAFLD nor other chronic liver diseases (*n* = 18,453,375) [9]. However, only hypertensive pregnancy complications, postpartum haemorrhage and prematurity were associated with MAFLD compared to those without on adjusted analysis (adjusted for age, ethnicity, parity and pre-existing metabolic disease) [9]. Notable limitations of these two studies are their retrospective nature, which could underestimate the true prevalence of MAFLD because it is not often screened for, or recorded in, national patient databases amongst young, reproductive-aged women, unless other metabolic risk factors are present.

To counteract the limitations of patient databases recording MAFLD status, we performed a large retrospective cohort study with our local hospital database and diagnosed MAFLD using clinical indices of hepatic steatosis, notably by calculating the HSI [73]. Of 11,929 multiethnic women with a singleton pregnancy, 1885 women had liver enzyme levels collected and an elevated HSI > 36 (suggestive of MAFLD) was identified in 1260 (66.8%) of them [73]. We identified that elevated HSI > 36 was associated with a composite of adverse maternal outcomes (GDM, gestational hypertension, pre-eclampsia or eclampsia; aOR 1.55, 95% CI 1.11–2.17, *p* = 0.01), but not a composite of adverse neonatal outcomes (pre-term birth, SGA or LGA neonates, neonatal hypoglycaemia or neonatal high-dependency unit admissions; aOR 1.17, 95% CI 0.94–1.45, *p* = 0.17) [73].

A secondary analysis of a multicentre prospective study from South Korea by Lee et al. [14] aimed to determine whether MAFLD led to increased adverse pregnancy outcomes (a composite of GDM, gestational hypertension, pre-term birth and congenital abnormalities) after studying 1744 pregnant Korean women in three groups of no NAFLD (*n* = 1523), NAFLD without evidence of metabolic dysfunction (*n* = 43) and MAFLD (*n* = 178) [20]. They identified that a higher rate of adverse pregnancy outcomes occurred in pregnant women with MAFLD than in those women without NAFLD (OR 2.16, 95% CI 1.52–3.07, *p* < 0.001) or those with NAFLD without metabolic dysfunction (OR 4.03, 95% CI 1.68–9.67, *p* < 0.002) [14]. Interestingly, the risk of adverse pregnancy outcomes in women with MAFLD remained significant despite adjusting for surrogate markers of insulin resistance (i.e., HOMA-IR, adiponectin and selenoprotein P levels), suggesting that the relationship between MAFLD and adverse pregnancy outcomes is likely mediated by other mechanisms than insulin resistance alone [14].

A systematic review and meta-analysis by El Jamaly et al. [19] included 22 studies, with a total of 13,641 women with MAFLD [19]. In regard to adverse maternal outcomes, when compared to pregnant women without MAFLD, those with MAFLD were more likely to have baseline hypertension (OR 3.75, 95% CI 2.13–6.59, *p* < 0.001), baseline diabetes (OR 6.0, 95% CI 2.21–16.31, *p* < 0.001), gestational hypertension (OR 1.83, 95% CI 1.03–3.26, *p* = 0.041), pre-eclampsia (OR 2.43, 95% CI 1.46–4.04, *p* = 0.001), a current diagnosis of GDM (OR 3.23, 95% CI 1.97–5.31, *p* < 0.001) or a prior history of GDM (OR 3.78, 95% CI 2.21–6.44, *p* < 0.001). Regarding adverse neonatal outcomes, pregnant women with MAFLD were more likely to experience premature delivery (OR 2.02, 95% CI 1.44–2.85, *p* < 0.001) or LGA neonates (OR 2.01, 95% CI 1.72–2.37, *p* < 0.001) compared to those without MAFLD [19].

Few studies have explored the consequences of the coexistence of MAFLD and GDM in pregnant women. Deng et al. [74] used FibroScan^®^ to identify MAFLD in pregnant women with an established diagnosis of GDM. MAFLD was diagnosed if the FibroScan^®^ CAP reading was ≥233.5 dB/m [74]. The prevalence of MAFLD in 108 pregnant women with diagnosed GDM from 24 weeks’ gestation onwards was 26.9% [74]. Of those, 18 (16.7%) women had mild hepatic steatosis (CAP cut-off score between 233.5 and 267 dB/m), 9 (8.3%) women had moderate hepatic steatosis (CAP cut-off score between 268 and 301 dB/m) and 2 (1.9%) women had severe hepatic steatosis (CAP cut-off score ≥ 302 dB/m) [74]. In comparison to those without MAFLD, women with MAFLD were more likely to require insulin therapy in the management of their GDM (31.6% vs. 62.1%; *p* = 0.004) [74].

Within a larger multiethnic cohort of pregnant women with GDM, incorporating participants from Deng et al.’s pilot study [74] (*n* = 380), we determined an even higher prevalence of FibroScan^®^-detected MAFLD at 38.7%, further suggesting that these two metabolic conditions are likely to be pathophysiologically entwined, together with overweight and obesity, as over half the study participants (67.6%) were overweight or obese [75]. Again, pregnant women with MAFLD and GDM were more likely to require insulin therapy (62.2% vs. 40.7%, *p* < 0.01), with a median peak insulin dosage of 10 units more than women without MAFLD [75]. Interestingly, women with GDM and MAFLD had comparable rates of adverse pregnancy outcomes to those with GDM without MAFLD [75]. While this finding does not suggest an additive effect of MAFLD on the presence of GDM, the study was not powered to assess for adverse pregnancy outcomes and the result may be confounded by the fact that recruitment for the study mostly occurred from attendees of a high-risk antenatal clinic, where close surveillance of women was already occurring. Further studies are required to explore whether the coexistence of MAFLD and GDM synergistically increases adverse maternal and neonatal outcomes.

### 5.4. Long-Term Maternal Consequences of MAFLD and GDM

Both MAFLD and GDM are metabolic conditions that individually predispose to a higher risk of cardiometabolic events [21,22,23,24,25].

Even without progression to T2D, women who have had a diagnosis of GDM are at a 2.3-fold increased risk of major cardiovascular events, either fatal or non-fatal ischaemic heart disease or cerebrovascular accidents in comparison to women without GDM (relative risk (RR) 2.31 [95% CI 1.57–3.39], *p* < 0.01) [22,76]. In particular, a meta-analysis of 10 studies with 1147 pregnant women with GDM and 7706 without GDM identified that GDM was independently associated with subclinical myocardial dysfunction, as assessed by speckle tracking echocardiography during pregnancy [77]. There is also a higher risk of traditional cardiovascular risk factors occurring in women with a prior history of GDM compared to those without GDM, with higher blood pressure readings, postpartum BMI, low-density lipoprotein cholesterol (LDL-C), triglycerides and decreased HDL-C concentrations observed as early as 12 months postpartum [22,78].

In addition, women with a previous history of GDM showed a significantly increased risk of developing venous thromboembolism compared to those without GDM (risk ratio 1.28, 95% CI 1.13–1.46) [79]. GDM is associated with a pro-inflammatory and pro-coagulatory state, with evidence of endothelial dysfunction and widespread systemic inflammation leading to platelet hyperactivity and eventual dysfunction [80]. Furthermore, hypoadiponectinaemia, which can occur in GDM, has been associated with venous thromboembolism [81] and was recently linked to neutrophil extracellular traps (NETs) formation, which may play a critical role in placental dysplasia [82].

MAFLD has recently been identified as a strong independent risk factor for cardiovascular disease by the American Heart Association (AHA) in their 2022 Scientific Statement [83]. Multiple traditional cardiovascular risk factors are present and increased in people with MAFLD, including obesity, T2D, hypertension and dyslipidaemia [83,84]. Independent of these risk factors, MAFLD remains associated with increased vascular endothelial inflammation, and early or subclinical atherosclerosis [83,84,85,86]. Further, in a systematic review and meta-analysis of 25,837 participants, Haddad et al. determined that people with MAFLD had a 1.8-fold higher risk of clinical cardiovascular events, defined as a composite of ischaemic heart disease, cerebrovascular accidents (excluding cerebral haemorrhage), cardiovascular-related deaths, symptomatic peripheral vascular disease and need for coronary intervention, in comparison to those patients without MAFLD (RR 1.77 [95% CI 1.26–2.48], *p* < 0.01) [87].

However, MAFLD increases the risk of not only arterial, but also venous thromboembolic disease [88]. In a case–control study of 138 patients with idiopathic venous thromboembolism, 112 (81%) patients were identified to have MAFLD by liver ultrasound [88,89]. MAFLD conferred an increased risk of developing venous thromboembolism even after adjustment for inherited thrombophilia (aOR 1.8, 95% CI 1.2–2.7, *p* < 0.0001) [89]. Prothrombotic mechanisms behind MAFLD are possibly multifactorial and not fully understood, but likely involve dysfibrinogenaemia, impaired fibrinolysis, endothelial dysfunction, increased production of factor VIII and von Willebrand factors and systemic inflammation with increased oxidative stress [88].

Although a transient condition, the long-term effects of GDM are significant and should not be underestimated. GDM has been established as a strong predictor for T2D, with the risk particularly increased amongst women affected by GDM if persistent fasting hyperglycaemia occurred in pregnancy, insulin therapy was required to treat GDM, an elevated BMI was present prior to pregnancy or if GDM recurred in subsequent pregnancies [90,91,92,93]. The overall RR for developing T2D after a GDM-affected pregnancy has been calculated as 6.0 (95% CI 4.1–8.8) [16]. Progression to T2D increases exponentially in the first five years after a GDM-affected pregnancy, before plateauing from 10 years onwards [93]. Interestingly, a prospective longitudinal cohort study by Gupta et al. in 200 South Asian women at a mean follow-up of 35 months postpartum suggests dysglycaemia might be more likely in women with both MAFLD and a prior history of GDM [94]. In this study, a significantly higher proportion of women with a prior history of GDM and MAFLD developed prediabetes or overt diabetes in comparison to those without either condition (43.8% vs. 24.2%, *p* = 0.048) [94]. However, the adjusted hazard ratio (aHR) was non-significant for new-onset prediabetes and T2D in those with MAFLD and GDM compared to those without either condition (aHR 1.99 [95% CI 0.80–4.96], *p* = 0.14), a result possibly attributed to their smaller sample size [94]. Nevertheless, the majority of women in this study were at a mean age of 32 ± 5 years at a median of 17 (8–39) months postpartum, and had already developed prediabetes or overt T2D at a young age [94], which is highly concerning.

The presence of a hyperglycaemic environment in utero may also play a significant role in the development of T2D in the offspring [95]. Prolonged exposure to intrauterine hyperglycaemia has been associated with a 5-fold increased risk of developing prediabetes and T2D [96], and a 2.5- to 4-fold increased risk of developing obesity and metabolic syndrome [97] amongst offspring born to GDM-affected women [96,97]. Maternal methylation secondary to GDM, combined with an adverse intrauterine environment, can synergistically contribute to deoxyribonucleic acid (DNA) methylation changes in the offspring [95,98]. Epigenetic changes arising in the fetus can lead to insulin and adipocyte dysregulation, pancreatic beta-cell dysfunction and impaired glucose metabolism, which have been associated with intergenerational development of insulin resistance and T2D [95]. Thus, an escalating intergenerational cycle of GDM, T2D, obesity and metabolic syndrome is occurring.

Recently, a systematic review and meta-analysis by Foo et al. included 12 studies that identified that infants or adolescents who were born to women with GDM had a 2-fold increased risk of developing MAFLD (OR 2.14, 95% GI 1.57–2.92) [99]. The development of MAFLD in offspring from GDM-affected mothers may be due to a few potential mechanisms that predispose the offspring to liver dysfunction at a younger age: 1. a GDM-complicated pregnancy can result in increased lipid flux through the placenta, causing placental dysfunction and increased maternal fatty acid circulation to the fetus and fetal liver; 2. maternal hyperglycaemia causing fetal hyperglycaemia and, in turn, fetal hyperinsulinaemia, both of which can escalate oxidative stress in the fetal liver and 3. maternal hyperglycaemia can lead to elevated circulating maternal fatty acid levels permeating through the placenta due to impaired glucose metabolism, with surplus fatty acids deposited in the fetal liver [99].

## 6. Management of Women with Both Metabolic Dysfunction-Associated Fatty Liver Disease and Gestational Diabetes Mellitus

### 6.1. Life Course Approach to Management

Lifestyle modification measures help mitigate the increased cardiometabolic risks associated with both MAFLD and GDM. There is now greater awareness of the benefits of adopting a life course approach to the management of GDM [22,38,100,101]. Typically viewed as a transient period of glucose intolerance, the current management approach for women with GDM predominantly focuses on intensive antenatal glycaemic management, in order to prevent short-term obstetric and neonatal complications [38,100,101]. However, GDM is a cardiometabolic disorder with implications that extend well beyond pregnancy [22,38,100,101]. This requires clinicians to monitor, prevent and minimise long-term maternal cardiometabolic risk by identifying and managing well-established cardiometabolic risk factors (i.e., dysglycaemia, dyslipidaemia, hypertension and obesity) in young women post-GDM [22,38,100,101].

The co-occurrence of MAFLD and GDM may signal an even higher risk trajectory towards developing long-term cardiometabolic complications, which should provide impetus for clinicians to monitor and aggressively manage modifiable risk factors early in these at-risk women. Key management priorities include the early implementation of lifestyle modifications, changing behavioural attitudes and minimising maternal gestational weight gain. Knowledge of future metabolic health risks also creates a unique opportunity for preventative long-term care, whereby women with both MAFLD and GDM may be more motivated to engage in and maintain healthy lifestyle behaviours in the immediate postpartum period and beyond, supported and actively encouraged by their healthcare providers.

Indeed, previous studies have determined that lifestyle changes, as implemented by various prevention programs in the postpartum period, can positively modify disease and risk factor trajectory [102,103,104,105]. Positive intervention outcomes include preventing T2D in women with a prior history of GDM, even up to 10 years post-diagnosis of GDM, via the Diabetes Prevention Program (DPP) [102,103], and reducing postpartum weight retention in women with a prior history of GDM with the Mothers after Gestational Diabetes in Australia Diabetes Prevention Program (MAGDA-DPP) [104], as well as the Gestational Diabetes’ Effects on Moms Diabetes Prevention Program (GEM-DPP) [105]. Whilst the focus of these postpartum intervention programs has been to reduce obesity and progression to T2D, it is likely they will have similar benefits on other cardiovascular risk factors, including MAFLD.

### 6.2. Increased Surveillance and Monitoring After Pregnancy Is Required for Women with Both MAFLD and GDM

To ensure a life course approach towards the management of women with both MAFLD and GDM, pregnancy should no longer be viewed as a transient state where metabolic complications can occur, but as a potential opportunistic life event where young women at high metabolic risk can be identified and further managed. Continued postpartum surveillance and the implementation of primary prevention strategies for cardiometabolic complications are required.

Several studies have evaluated the use of postpartum transitional care clinics for women who experienced hypertensive disorders in pregnancy and were at risk of developing cardiometabolic complications [106,107,108,109]. Most of these postpartum transitional care clinics were staffed by a multidisciplinary team, including an obstetric medicine physician, cardiologist, nurse practitioner and dietitian [106,107,108]. Duration of follow-up ranged between 6 months [106,107] and 5 years [108]. Benefits of a postpartum transitional care clinic included ongoing education and assessment of cardiometabolic risk [106], continued encouragement and support for lifestyle interventions in the postpartum period [107] and optimal engagement and transition of longitudinal surveillance and management to primary care physicians for these at-risk women [106,107,108].

While benefits from transitional care clinics have been demonstrated, follow-up in an outpatient postpartum transitional hospital clinic or involvement in postpartum prevention programs have proven to be difficult [109]. A major limitation is the suboptimal retention rates [104,105,106,107,108,109]. The reasons for this are likely multifactorial, including mothers being time poor, with many competing family demands mainly revolving around the care of their newborn, the lack of virtual or telehealth options for follow-up care and the distance involved in travelling to a hospital clinic for in-person attendance [109]. Further, there are substantial limitations in scalability due to significant financial challenges and intensive resources involved in sustaining a multidisciplinary postpartum transitional clinic long term, with suboptimal participation from the target patient group [109].

If the goal is ultimately to ensure that the initial screening and long-term management of cardiometabolic risk factors for at-risk women are embedded into routine primary care [22], then it may be more effective to focus on the effective transition of these at-risk women to primary healthcare providers soon after an affected pregnancy. Primary care physicians are well placed to continue preventative health measures once women are identified as having MAFLD and GDM during pregnancy, given the close rapport and easier access to follow-up appointments [110]. In general, primary care physicians possess a positive attitude towards selective cardiometabolic risk assessment and ongoing management for at-risk individuals, and consider it as part of their responsibility [111].

However, there is a need for more guidance, particularly in the formulation of a standardised risk assessment and a systematic approach to the screening and management of cardiometabolic risk factors in these young, reproductive-aged women in both the immediate postpartum period and long term [22]. The first step would be to increase clinician awareness regarding the importance of a life course approach to the management of these high-risk women identified as having both MAFLD and GDM during pregnancy.

## 7. Conclusions

The rising global prevalence of MAFLD and GDM has established the clinical importance of both of these metabolic conditions [1,38], particularly with regard to their associated adverse maternofetal consequences [9,17,18,19,20]. In particular, the likely adverse synergistic relationship between MAFLD and GDM warrants further exploration. Future prospective longitudinal cohort studies are required to determine whether the concomitant presence of MAFLD and GDM synergistically increases maternal and neonatal adverse events, and whether selective MAFLD screening, particularly in those at high metabolic risk, is of benefit in helping develop early preventative and management strategies for these women.

The diagnosis of both MAFLD and GDM in young, reproductive-aged women provides a unique opportunity to offer further education and risk management of both metabolic conditions, particularly in initiating effective lifestyle interventions at an earlier stage. It is important to prevent the increased cardiometabolic risk associated with MAFLD and GDM from developing into overt cardiovascular complications for these young, at-risk women, while also preventing the metabolic intergenerational consequences for their offspring.

## Figures and Tables

**Table 1:** Studies evaluating the relationship between a prior history of GDM and postpartum MAFLD development.

Study (Ref.)	Study Participants (*n*)	Postpartum Follow-Up (Years)	Modality Used for MAFLD Diagnosis	GDM Diagnostic Criteria (mmol/L)	Risk of MAFLD Development aOR or aHR [95% CI], *p*-Value	Variables Adjusted for	Other Significant Variables Associated with MAFLD
Forbes et al., 2011 [60]	*pGDM*: 110 *Control*: 113	1–10	• Liver USS	75 g OGTT: FBGL ≥ 7.0 2 h BGL ≥ 7.8	aOR 2.77 [1.43–5.37], *p* = 0.002	BMI	HOMA insulin sensitivity: aOR 0.08 [95% CI 0.04–0.19] per 1 SD, *p* < 0.001 ↑ ALT: aOR 2.85 [95% CI 1.63–4.98] per 1 SD, *p* < 0.001
Foghsgaard et al., 2017 [61]	*pGDM*: 100 *Control*: 11	Median 4.5–4.8	• Liver USS	75 g OGTT: • 2 h BGL ≥ 7.8	Not assessed (MAFLD prevalence 22% (*n* = 24) in women with a pGDM)	Not assessed	Insulin resistance (Matsuda Index): aOR 0.44 [95% CI 0.23–0.75], *p* = 0.0057 Waist circumference: aOR 1.07 [95% CI 1.02–1.12], *p* = 0.0109
Mehmood et al., 2018 [62]	*pGDM*: 97 *pGIGT*: 40 *Control*: 120	Mean 4.8	• Liver USS	100 g OGTT: FBGL ≥ 5.8 1 h BGL ≥ 10.6 2 h BGL ≥ 9.2 3 h BGL ≥ 8.1	aOR 3.66 [1.1–12.5], *p* = 0.04 ^1^	Age, ethnicity, family history of diabetes, BMI, breast feeding duration, GDM/GIGT, liver fat score ≥ 2	Not assessed
Ajmera et al., 2016 [63]	*pGDM*: 124 *Control*: 991	25	• Non-contrast CT ^2^	Self-report	aOR 2.29 [1.23–4.27], *p* = 0.01	Age, parity, baseline BMI, waist circumference, HOMA-IR, HDL-C, triglycerides	Baseline HOMA-IR: aOR 1.56 [95% CI 1.20–2.04], *p* < 0.01 Baseline triglycerides: aOR 1.05 [95% CI 1.01–1.11], *p* = 0.03
Cho et al., 2023 [64]	*pGDM*: 4683 *Control*: 59,714	Median 3.7	• Liver USS	Self-report	aHR 1.46 [1.33–1.59] (overall MAFLD); *p* not reported aHR 1.75 [1.25–2.44], (moderate-to-severe MAFLD); *p* not reported	Current age, age at first birth, hospital centre, BMI, examination year, alcohol consumption, smoking status, physical activity, education level, medication use for hyperlipidaemia, history of hypertension, CVD	T2D-mediated interaction between pGDM and overall MAFLD development: excess RR 0.01 [95% CI −0.01 to 0.03] HOMA-IR-mediated interaction between pGDM and overall MAFLD development: excess RR 0.005 [95% CI −0.003 to 0.012]

^1^ pGDM and pGIGT women analysed together against healthy control (NGT) group. ^2^ MAFLD diagnosed if liver attenuation ≤ 40 HU. aHR: adjusted hazard ratio; aOR: adjusted odds ratio; ALT: alanine aminotransferase; BGL: blood glucose level; BMI: body mass index; CI: confidence intervals; CT: computed tomography; CVD: cardiovascular disease; FBGL: fasting blood glucose level; g: gram; GDM: gestational diabetes mellitus; GIGT: gestational impaired glucose tolerance; HDL-C: high-density lipoprotein cholesterol; HOMA-IR: homeostatic model assessment for insulin resistance; HOMA: homeostatic model assessment; h: hour; MAFLD: metabolic dysfunction-associated fatty liver disease; *n*: number; NGT: normal glucose tolerance; OGTT: oral glucose tolerance test; *p*: *p*-value (significant at <0.05); pGDM: prior history of gestational diabetes mellitus; pGIGT: prior history of gestational impaired glucose tolerance; RR: relative risk; SD: standard deviation; T2D: type 2 diabetes mellitus; USS: ultrasound; ↑: increase.

**Table 2:** Studies evaluating the relationship between the detection of MAFLD in pregnancy and development of GDM.

Study (Ref.)	Study Participants (*n*)	Predominant Ethnicity (*n*, %)	Modality Used for MAFLD Diagnosis	Gestation (Weeks) of MAFLD Diagnosis	GDM Diagnostic Criteria (mmol/L)	Risk of GDM Development aOR [95% CI], *p*-Value	Variables Adjusted for
De Souza et al., 2016 [11]	*Total*: 476 *MAFLD*: 77	• Caucasian (247, 52)	• Liver USS	11–14	75 g OGTT at 24–28 weeks: FBGL ≥ 5.3 1 h BGL ≥ 10.6 2 h BGL ≥ 8.9	• 2.2 [1.1–4.3], *p* not reported	Age, ethnicity, family history of T2D, BMI (pre-gravid), BMI (changed), ≥1 USS feature of hepatic fat
Mousa et al., 2018 [65]	*Total:* 400 *MAFLD*: 200	• Egyptian (400, 100)	• Liver USS	8–12	75 g OGTT at 24–28 weeks: FBGL ≥ 7.0 2 h BGL ≥ 11.1	Not assessed	Not assessed
Lee et al., 2019 [12]	*Total*: 608 *MAFLD*: 112	• Korean (608, 100)	Liver USS FLI HSI	10–14	50 g OGTT GCT at 24–28 weeks: • 1 h ≥ 7.8 If positive GCT, then 100 g OGTT performed ^1^: FBGL ≥ 5.3 1 h BGL ≥ 10.0 2 h BGL ≥ 8.6 3 h BGL ≥ 7.8	Liver USS: 2.5 [1.1–5.8], *p* < 0.05 FLI: 1.1 [1.0–1.1], *p* < 0.005 HSI: 1.1 [1.0–1.2], *p* < 0.05	Age, pGDM, waist circumference, SBP/DBP, HOMA-IR, Selenoprotein P levels > 34.01 μg/mL, Adiponectin levels <1.72 μg/mL
Deng et al., 2021 [66]	*Total*: 50 *MAFLD*: 6	South Asian (26, 52) East and Southeast Asian (16, 32)	• Transient elastography (FibroScan^®^) ^2^	<24	75 g OGTT at 24–28 weeks: FBGL ≥ 5.5 2 h BGL ≥ 8.0	Not assessed	Not assessed
Herath et al., 2019 [67]	*Total*: 573 *MAFLD*: 104	• Sinhala (542, 95)	• Liver USS	30+	FBGL ≥ 5.1 at 24–28 weeks or 75 g OGTT at 24–28 weeks: FBGL ≥ 5.1 1 h BGL ≥ 10.0 2 h BGL ≥ 8.5	• 1.3 (0.8–2.3), *p* not reported	Age, BMI, gestational HTN, pre-eclampsia, ≥1 grade steatosis on liver USS
Sattari et al., 2020 [68]	*Total*: 84 *MAFLD*: 12	• Caucasian (51, 61)	• Liver USS	30+	75 g OGTT at 24–28 weeks: FBGL ≥ 5.1 1 h BGL ≥ 10.0 2 h BGL ≥ 8.5	Not assessed	Not assessed

^1^ A positive diagnosis for GDM was obtained if ≥2 elevated BGLs were obtained on the 100 g OGTT. ^2^ MAFLD diagnosed if cut-off controlled attenuation parameter (CAP) ≥ 233.5 dB/m. aOR: adjusted odds ratio; BGL: blood glucose level; BMI: body mass index; CAP: controlled attenuation parameter; CI: confidence intervals; DBP: diastolic blood pressure; FBGL: fasting blood glucose level; FLI: Fatty Liver Index; g: gram; GCT: glucose challenge test; HOMA-IR: homeostatic model assessment for insulin resistance; h: hour; HSI: Hepatic Steatosis Index; HTN: hypertension; MAFLD: metabolic dysfunction-associated fatty liver disease; *n*: number; OGTT: oral glucose tolerance test; pGDM: prior history of gestational diabetes mellitus; SBP: systolic blood pressure; T2D: type 2 diabetes mellitus; USS: ultrasound.

## Abbreviations

The following abbreviations are used in this manuscript:

AHA	American Heart Association
aHR	Adjusted hazard ratio
aOR	Adjusted odds ratio
aRR	Adjusted risk ratio
BGL	Blood glucose level
BMI	Body mass index
CAP	Controlled attenuation parameter
CARDIA	Coronary Artery Risk Development in Young Adults
CI	Confidence interval
CT	Computed tomography
DNA	Deoxyribonucleic acid
DPP	Diabetes Prevention Program
DSP	Diastolic blood pressure
EASD	European Association for the Study of Diabetes
EASL	European Association for the Study of Liver
EASO	European Association for the Study of Obesity
FBGL	Fasting blood glucose level
FLI	Fatty Liver Index
GCT	Glucose challenge test
GDM	Gestational diabetes mellitus
GEM-DPP	Gestational Diabetes’ Effects on Moms Diabetes Prevention Program
HAPO	Hyperglycaemia and Adverse Pregnancy Outcome
HDL-C	High-density lipoprotein cholesterol
HOMA-IR	Homeostatic model assessment for insulin resistance
h	Hour
HSI	Hepatic Steatosis Index
HTN	Hypertension
HU	Hounsfield unit
IADPSG	International Association of Diabetes and Pregnancy Study Groups
ICD	International Classification of Diseases
IDF	International Diabetes Federation
IFG	Impaired fasting glucose
IGT	Impaired glucose tolerance
LDL-C	Low-density lipoprotein cholesterol
LGA	Large-for-gestational age
LSM	Liver stiffness measurement
MAFLD	Metabolic dysfunction-associated fatty liver disease
MAGDA-DPP	Mothers after Gestational Diabetes in Australia Diabetes Prevention Program
MASLD	Metabolic dysfunction-associated steatotic liver disease
NAFLD	Non-alcoholic fatty liver disease
NETs	Neutrophil extracellular traps
NGT	Normal glucose tolerance
NHANES III	Third National Health and Nutrition Examination Survey
NIS	National Inpatient Sample
OGTT	Oral glucose tolerance test
OR	Odds ratio
PCOS	Polycystic ovarian syndrome
pGDM	Prior history of gestational diabetes mellitus
pGIGT	Prior history of gestational impaired glucose tolerance
RCT	Randomised controlled trial
RR	Relative risk
SBP	Systolic blood pressure
SGA	Small-for-gestational age
SMBG	Self-monitoring of blood glucose
T2D	Type 2 diabetes mellitus
USA	United States of America
USS	Ultrasound
